# Deep learning detection of retinitis pigmentosa inheritance forms through synthetic data expansion of a rare disease dataset

**DOI:** 10.1038/s41598-026-47341-9

**Published:** 2026-04-11

**Authors:** Elizabeth E. Hwang, Max L. Rivera, Man Ting Lin, Pierre Zéboulon, Krish Nachnani, Olivia Yuan, Pulkit Madaan, Ying Han, Jacque L. Duncan, Lin Jia, Jing Shan

**Affiliations:** 1 Department of Ophthalmology, University of California, San Francisco, San Francisco, CA USA; 2 Digillect LLC, San Francisco, CA USA; 3 Department of Corneal and refractive surgery, Rothschild Foundation Hospital, Paris, France

**Keywords:** Computational biology and bioinformatics, Diseases, Genetics, Medical research

## Abstract

Classification of inheritance patterns is important for clinical characterization and genetic counseling in inherited retinal diseases (IRDs). In practice, inheritance assessment integrates pedigree information, clinical evaluation, and genetic testing. However, a definitive molecular diagnosis is not achievable in a subset of patients, even with contemporary sequencing approaches, and family history may be incomplete or ambiguous. These limitations motivate investigation of complementary phenotype-based approaches that may provide additional contextual information, while not replacing molecular diagnosis when available. Deep learning (DL) applied to fundus imaging presents a promising approach for automated inference of inheritance modes, as recent advances in oculomics have demonstrated applications of DL in uncovering subtle phenotypic patterns associated with retinal conditions. However, development has been hindered by the low prevalence of IRDs and the scarcity of annotated datasets in individual clinical settings. In this study, we focus on retinitis pigmentosa (RP), a highly heterogeneous disorder in both clinical presentation and genetic etiology. We present a first-in-class deep learning approach that leverages Vision Transformer (ViT) models to distinguish autosomal from X-linked RP using color fundus photography. To overcome challenges posed by limited data, we introduce an innovative variational autoencoder–based data expansion strategy, which improves inheritance pattern classification based on color fundus photos from 0.67 AUC to 0.79 AUC. Our findings demonstrate the potential of deep learning to uncover subtle phenotypic differences linked to genetic inheritance, complementing existing genetic testing approaches, and introduce a novel training data augmentation method to render deep learning accessible to rare diseases.

## Introduction

For rare inherited retinal diseases (IRDs), determining the mode of inheritance (i.e. autosomal versus X-linked inheritance) is important for providing accurate genetic counseling, guiding family planning, and predicting disease progression. In a subset of cases, inheritance and genetic etiology remain unresolved, and may be due to a variety of factors such as multigenic inheritance and variable expressivity/penetrance^1,2^. The most accessible and cost-effective option, whole-exome sequencing (WES), focuses on the protein-coding region, but coverage is limited and may miss disease-causing variants in non-coding regions^3,4^. Though more comprehensive, whole-genome sequencing (WGS) has other limitations, such as difficulty in detecting certain types of variants^5^. Furthermore, the diagnostic yield of next-generation sequencing for patients with RP ranges from 50 to 75%^6,7^, and approximately half of non-syndromic RP patients are cases with no family history of disease, which complicates the determination of inheritance patterns^8^. In such contexts, complementary imaging may provide additional contextual information, as previous research indicated that distinct genetic inheritance patterns may result in subtle morphological features that are unfortunately challenging to detect by the human eye alone^9,10^.

Artificial intelligence (AI) enhanced imaging tools may offer a complementary, non-invasive source of phenotypic information. In clinical medicine, contemporary deep learning (DL) models have achieved disease diagnostic accuracies comparable to those of experienced physicians^11–13^. More remarkable, some DL models can further discern sub-visual features—such as inferring biological sex or age from fundus photographs—that elude human observers^14–16^. Recently, deep learning model Eye2Gene trained on multimodal imaging from thousands of patients has revealed predictive values of causative genes for inherited retinal diseases^17^. This success, however, has come at the cost of prodigious data demands. In response, the field is converging on foundation-model strategies—both general and task-specific—that aim to improve resource efficiency and cross-task generalization^18^. General-purpose vision encoders typically require 142–300 million heterogeneous images to attain competitive performance^19,20^, whereas task-tailored variants achieve state-of-the-art (SOTA) accuracy with 1.6–3.4 million curated ophthalmic images^13,21,22^. Transfer learning techniques can further shrink data requirements by orders of magnitude to 70,000 to 100,000 images^23,24^. While these AI techniques have empowered detection and grading of common retinal conditions^25,26^, such as diabetic retinopathy (DR)^12^ and age-related macular degeneration (AMD)^27^, reduced data thresholds still pose a prohibitive barrier for fields like IRDs, where annotated datasets seldom exceed a few hundred cases owing to low prevalence and fragmented data stewardship^28^.

One promising strategy for curbing data demands is to leverage generative AI, which can augment existing datasets with high-fidelity synthetic images^29–31^. Recent work has demonstrated the feasibility and utility of generative models for synthetic data generation in medical and biomedical imaging, including applications in radiology^32,33^. Synthetic data has been generated and utilized in multiple settings that are challenged by scarce datasets, including the development of multiracial facial recognition models, the augmentation of chest X-ray dataset, and the curation of customized organ models for surgical simulation and training^34–36^. Among the generative approaches, diffusion-based models recently achieved strong perceptual quality and have been increasingly explored for medical image synthesis^37^. Generative adversarial networks (GANs) represent another widely adopted paradigm for image synthesis and have demonstrated good performance across a broad range of medical imaging applications, including radiology and ophthalmology^33,38^. While showing great potential, the performance and stability of many generative AI methods, particularly diffusion-based models, are sensitive in severely data-limited settings; they can be prone to hallucination, where the produced outputs are too far detached from reality, creating synthetic images that are plausible but nonsensical^39,40^. To address this, we explored the use of a variational autoencoder (VAE). VAE is a generative model that learns to encode input data into a latent space defined by a probability distribution, typically a multi-variate Gaussian^41^. During training, the model optimizes a loss function that balances reconstruction accuracy with regularization, ensuring the latent space conforms to a known prior distribution. To generate synthetic data, new samples are drawn from this prior distribution and passed through the decoder network to produce novel but statistically consistent outputs^42^. By generating outputs that adhere to specific input data, VAE-based approaches can offer greater controllability over generated outputs in certain settings, which may help reduce severe forms of hallucination relative to unconstrained generative methods.

Previously, we demonstrated that VAE-enhanced synthetic datasets significantly improved glaucoma detection by ViT^30^. Here we report on the development of a second-generation VAE-based data enhancement workflow to deliver dataset diversity beyond previous methods and explore how this new functionality can help improve the feasibility of applying DL methods to rare diseases.

## Methods

### Patient cohort

Fundus photographs were acquired from patients seen at University of California San Francisco between October 2018 and August 2024 with a familial and/or sequencing-confirmed diagnosis of non-syndromic retinitis pigmentosa, including autosomal dominant (AD), autosomal recessive (AR), X-linked recessive (XR), or patients with sequencing-confirmed X-linked carrier (XLC) status. A total of 134 color fundus photographs were included in the full dataset. Symptom duration was determined from chart review by a retinal specialist with IRD expertise (J.L.D.), and was defined as the length of time between patient-reported onset of visual symptoms and imaging date, rounded up to the nearest year. Asymptomatic patients were assigned a symptom duration of 0 years. Statistical analysis was performed with GraphPad Prism software (10.0.3). This study was conducted in accordance with the tenets of the Declaration of Helsinki. Ethical approval was obtained from the Institutional Review Board (IRB) of the University of California, San Francisco (UCSF; IRB approval number: 21–35673). As this was a retrospective study involving no more than minimal risk to participants and no practicable means of obtaining consent, the UCSF IRB determined that a waiver of informed consent was appropriate.

### Data preprocessing

For the purpose of confirming laterality, only fundus photos with clearly visible macula and optic nerve head (ONH) structures were included. If the patient had multiple imaging dates, only the most recent date was included for review. Fundus photos from the most recent date were manually assessed for image quality (excessive blur, artifact, sufficient field of view). In addition, all right eye (OD) images were horizontally flipped to match the image orientation of the left eye (OS) prior to model training. Eyes without images meeting these quality criteria were excluded.

### Synthetic data generation

We evaluated three synthetic image generation paradigms as candidate strategies for training set enhancement: Generative Adversarial Networks (GAN), diffusion, and variational autoencoders (VAE). Diffusion synthesis used runwayml/stable-diffusion-v1-5 in an image-to-image setup (denoising strength = 0.5; 50 inference steps). For GAN-based generation, we implemented a Deep Convolutional GAN (DCGAN) with a 100-dimensional latent vector and trained it to produce 224 × 224 RGB fundus photographs^43^. For VAE-based generation, we tested two latent-space expansion schemes: (i) **Gen 1**, random-noise perturbations of latent embeddings; and (ii) **Gen 2**, pairwise combinatorial expansion by mixing latent representations of image pairs. We compared these approaches using Fréchet Inception Distance (FID) and by examining the distributional alignment of latent embeddings between real and synthetic data. VAE was selected as the platform for synthetic data generation in subsequent experiments.

#### Random noise expansion (Gen 1) VAE

Synthetic images were generated by introducing noise into the latent space using four noise distributions: constant, Gaussian, uniform, and sinusoidal. For each image, one strategy was randomly selected and applied with a randomly sampled strength parameter from 0.05 to 1 to the embedding, leading to additional variations in the output images. Each image along with their random noise expansion was added to the training set resulting in a two-fold expansion of the training data. We utilized a dual-level ResNet-based encoder-decoder structure trained on ImageNet with pixel-wise reconstruction loss.

#### Pairwise combinatorial expansion (Gen 2) VAE

**Fig. 1:**
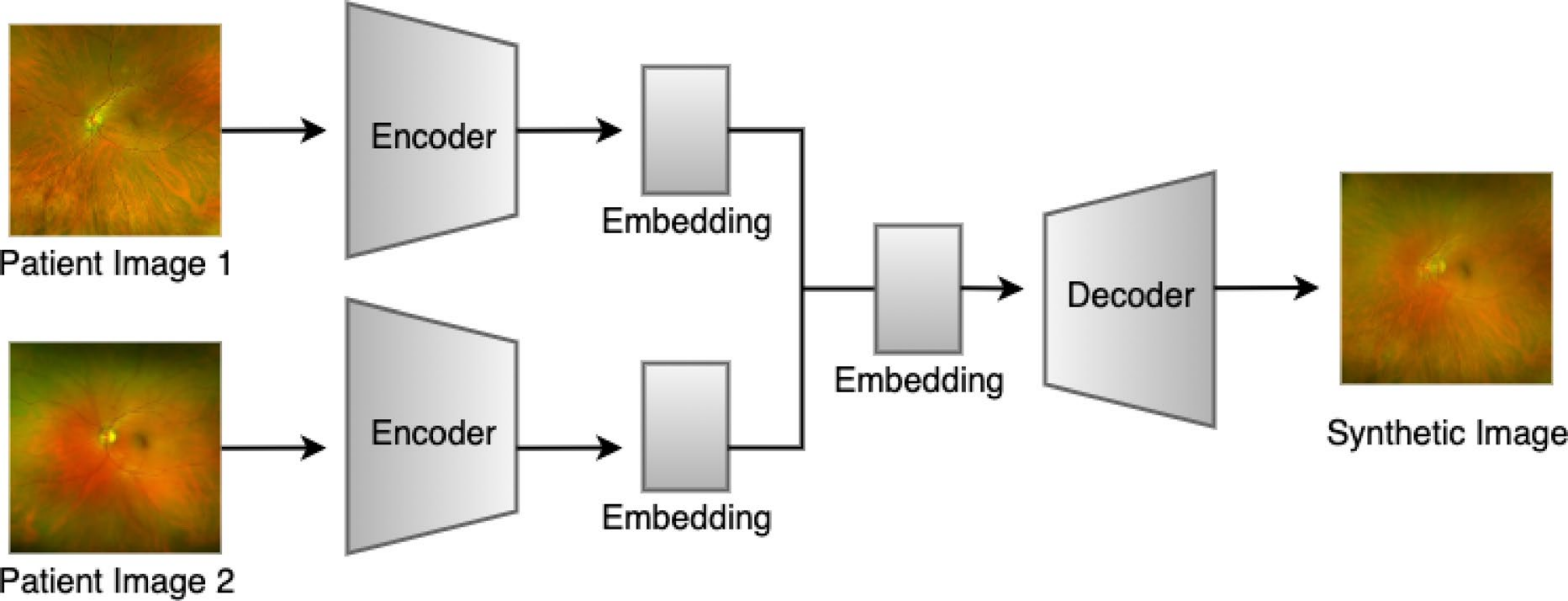
Two-way combinatorial synthetic image generation using variational autoencoder.

**Fig. 2:**
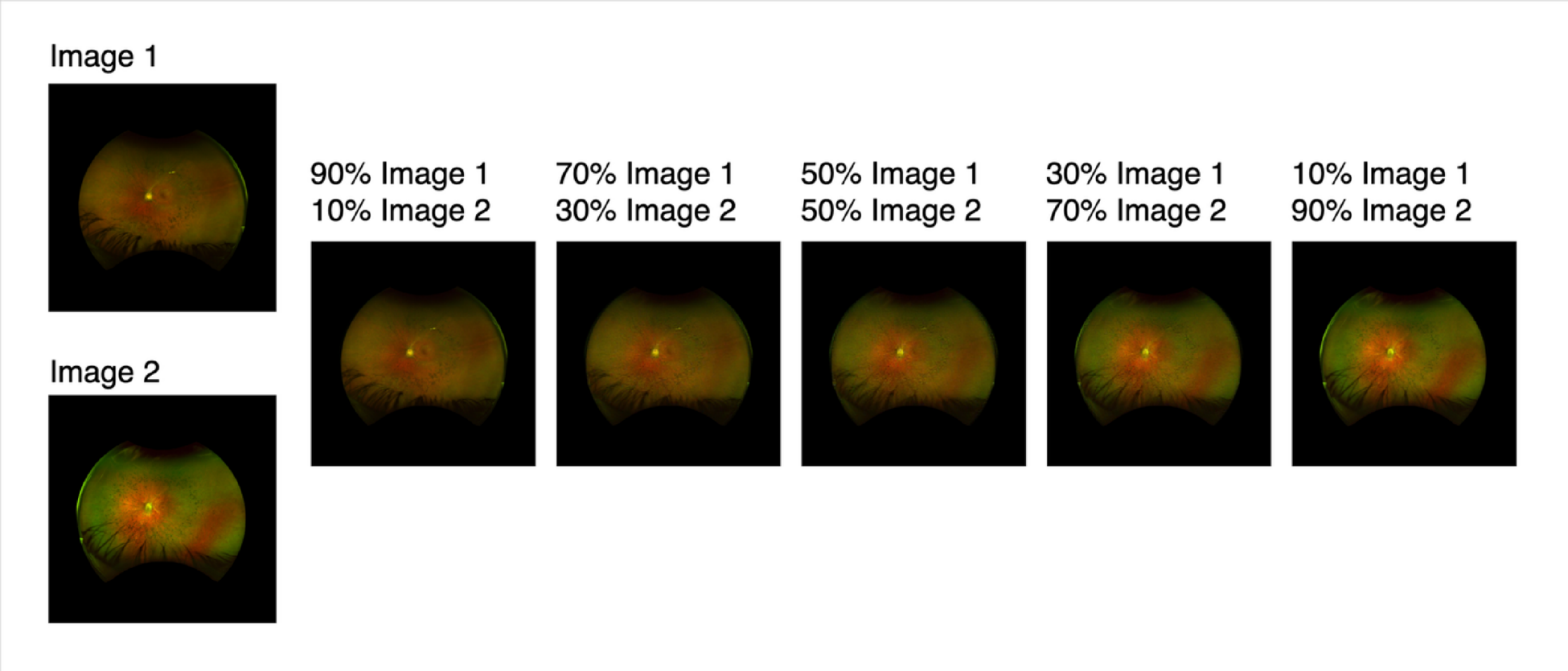
Examples of Synthetic Images based on Autosomal Dominant (AD) RP images. Left to right A/B ratios: 0.1, 0.3, 0.5, 0.7, 0.9.

To expand data enhancement capabilities in terms of both quantity and diversity, we developed a 2nd generation framework to perform pairwise combinatorial expansions using VAE. Here, synthetic images were generated by combining the latent representations of every possible pair of training images that share the same genotype label (Fig. 1). This pairwise structure ensures that every unique two-image combination within a genotype class contributes to the synthetic dataset. For each image pair, we encoded both images using a pretrained variational autoencoder (AutoencoderKL) and linearly combined their latent vectors using a predefined set of mixing ratios of 0.1, 0.3, 0.5, 0.7, and 0.9, as illustrated in Fig. 2. Each mixing ratio governs the relative contribution of each image’s latent representation to the resulting composite embedding. This process is repeated for n x n combinations for each label, where n is the number of images with the same label, augmenting each inheritance mode by C (n, 2) images. The resulting composite latent vector is then decoded to produce a synthetic image.

### Classification model selection

To assess architectural suitability for inheritance-pattern classification on fundus images, we compared a convolutional neural network (Microsoft ResNet-50) with a transformer-based model (Vision Transformer, ViT). Both networks were initialized with pretrained weights from the ImageNet dataset and fine-tuned using the AdamW optimizer with a linear learning rate schedule. Models were evaluated in two regimes: (i) training on the original dataset without synthetic augmentation and (ii) training on datasets expanded with synthetic images. We applied an 80/20 train-test data split, ensuring that the fundus images of both eyes from the same patient are incorporated into the same split. Comparative performance was reported (AUC, accuracy, recall and specificity) on the same held-out test set to quantify the impact of architecture choice. Based on these metrics, ViT was selected as the platform for detection of RP inheritance forms in subsequent experiments.

### Vision transformer training and evaluation

**Fig. 3:**
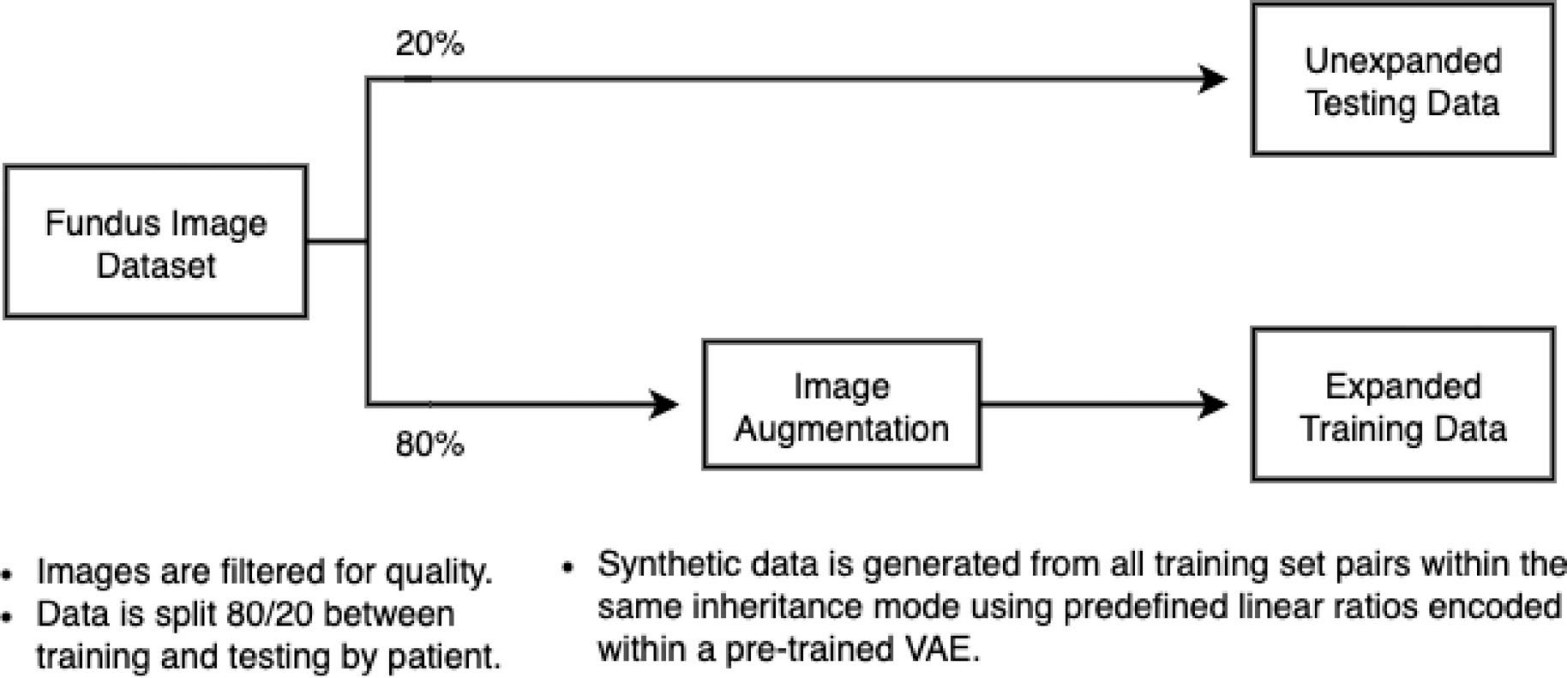
Workflow for Data Expansion and Vision Transformer Model Training. *VAE* Variational Autoencoder, *ViT* Vision Transformer.

The pretrained foundation model used in this study (Google’s vit-base-patch16-224-in21k) was initialized with pretrained weights from ImageNet-21k. All images were resized to 224 × 224 pixels, following established preprocessing protocols for ViT^44,45^. Training was performed for 30 epochs, using the AdamW optimizer with a learning rate of 5e-05. During training, we applied data augmentations using a randomized resizing crop followed by a randomized horizontal flip, introducing variability and reducing overfitting. Cross validation was performed by re-sampling to generate representative train/test splits. Synthetic images were generated from and added to only the training sets. For inheritance pattern detection, we trained two ViT models: one using only the original fundus images and the other using synthetically expanded training data (Fig. 3). A third ViT model was trained in a subsequent 3-class variation to assess the model’s ability to distinguish age-matched normal controls vs. autosomal RP eyes vs. x-linked RP eyes. Class sizes were balanced to minimize prevalence bias. Mean accuracy, recall, and specificity were averaged over five-fold cross-validation. Pooled AUCs were calculated by aggregating labels and predictions generated across all validation sets.

## Results

### Retinitis pigmentosa (RP) patient demographics

**Fig. 4:**
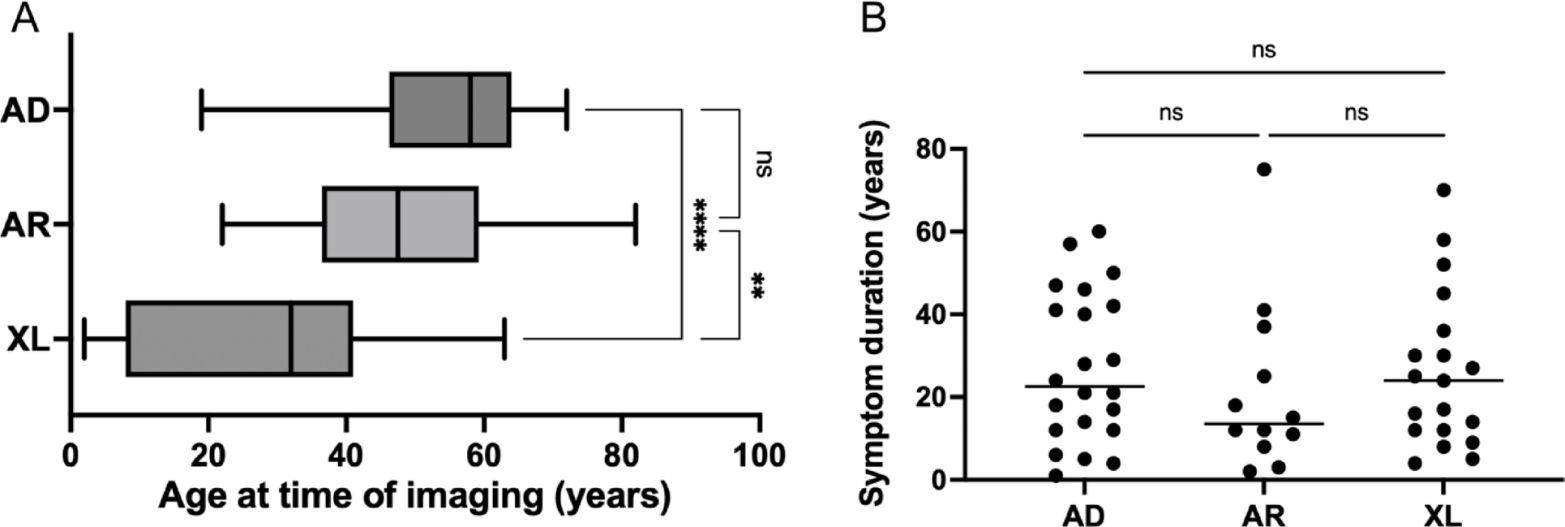
Patients mean age (panel **A**) and median symptom duration at time of imaging (panel **B**). *AD* Autosomal Dominant, *AR* Autosomal Recessive, *XL* X-linked Recessive and X-linked Carrier (combined for anonymization purposes). ** denotes p-value < 0.005 and *** denotes p-value < 0.0001.

Our final cohort included 105 eyes from 53 retinitis pigmentosa (RP) patients, for a total of 134 wide-field color fundus photos (Table 1). X-linked recessive (XR) and X-linked carrier (XLC) patient characteristics were reported as a single category to protect confidentiality. Patients’ mean age at time of imaging was 53 years (± 16 years) for autosomal dominant (AD), 49 years (± 17 years) for autosomal recessive (AR), and 26 years (± 19 years) for XR or XLC (Table 1; Fig. 4). Mean patient ages were significantly different (ordinary one-way ANOVA with Tukey’s multiple comparisons test, p-value < 0.01) between the autosomal (AD, AR) and X-linked (XL) groups, in line with known earlier onset of symptoms in X-linked RP^46^. Patient median symptom duration at time of imaging was 27 years (± 18 years) for AD, 22 years (± 21 years) for AR and 26 years (± 19 years) for XL, with no significant inter-group differences by one-way ANOVA (Table 1; Fig. 4).

**Table 1:** Study cohort. *AD* Autosomal Dominant, *AR* Autosomal Recessive, *XL* X-linked Recessive and X-linked Carrier (combined for anonymization purposes).

	AD	AR	XL	All
Patients	22	13	19	54
Sex				
Males	11	9	12	32
Females	11	4	7	22
Median symptom duration in years (SD)	27 (18)	22 (21)	26 (19)	
Mean age at time of imaging in years (SD)	53 (16)	49 (17)	26 (19)	

### Classification model selection

Model performance was compared between a ResNet convolutional neural network and a vision transformer (ViT) architecture on both the original dataset and the synthetically expanded dataset (Gen 2) (Table 2). On the base dataset, the two architectures demonstrated comparable performance, with ViT achieving a slightly higher AUC (0.70) than ResNet (0.68). Following dataset expansion, performance improvements were observed for both models; however, the gain was more pronounced for the ViT architecture. ViT trained on the Gen 2 - expanded dataset achieved the highest performance overall, with a pooled AUC of 0.79 and accuracy of 0.71. In comparison, the ResNet model trained on the expanded dataset achieved a pooled AUC of 0.67 and accuracy of 0.66. These results indicate that while both architectures benefit from synthetic data expansion, the ViT architecture demonstrates a larger performance improvement under enhanced training conditions.

**Table 2:** Performance comparison of ResNet and ViT, each trained on base (unexpanded images) and Gen2-expanded datasets. Standard deviation ranges are bracketed (−1 SD, + 1 SD).

Model Performance						
Model	Dataset	AUC	Pooled AUC	Accuracy	Recall	Specificity
ResNet	Base	0.68 (0.54, 0.81)	0.63	0.61 (0.55, 0.67)	0.48 (0.47, 0.50)	0.97 (0.94, 1.00)
ResNet	Gen 2	0.69 (0.58, 0.80)	0.67	0.66 (0.60, 0.71)	0.64 (0.54, 0.73)	0.67 (0.54, 0.81)
ViT	Base	0.70 (0.64, 0.76)	0.67	0.62 (0.57, 0.67)	0.55 (0.48, 0.62)	0.55 (0.48, 0.62)
ViT	Gen 2	0.79 (0.68, 0.90)	0.79	0.71 (0.61, 0.81)	0.68 (0.58, 0.78)	0.68 (0.58, 0.78)

### Vision transformer classification of disease inheritance mode in RP

**Fig. 5:**
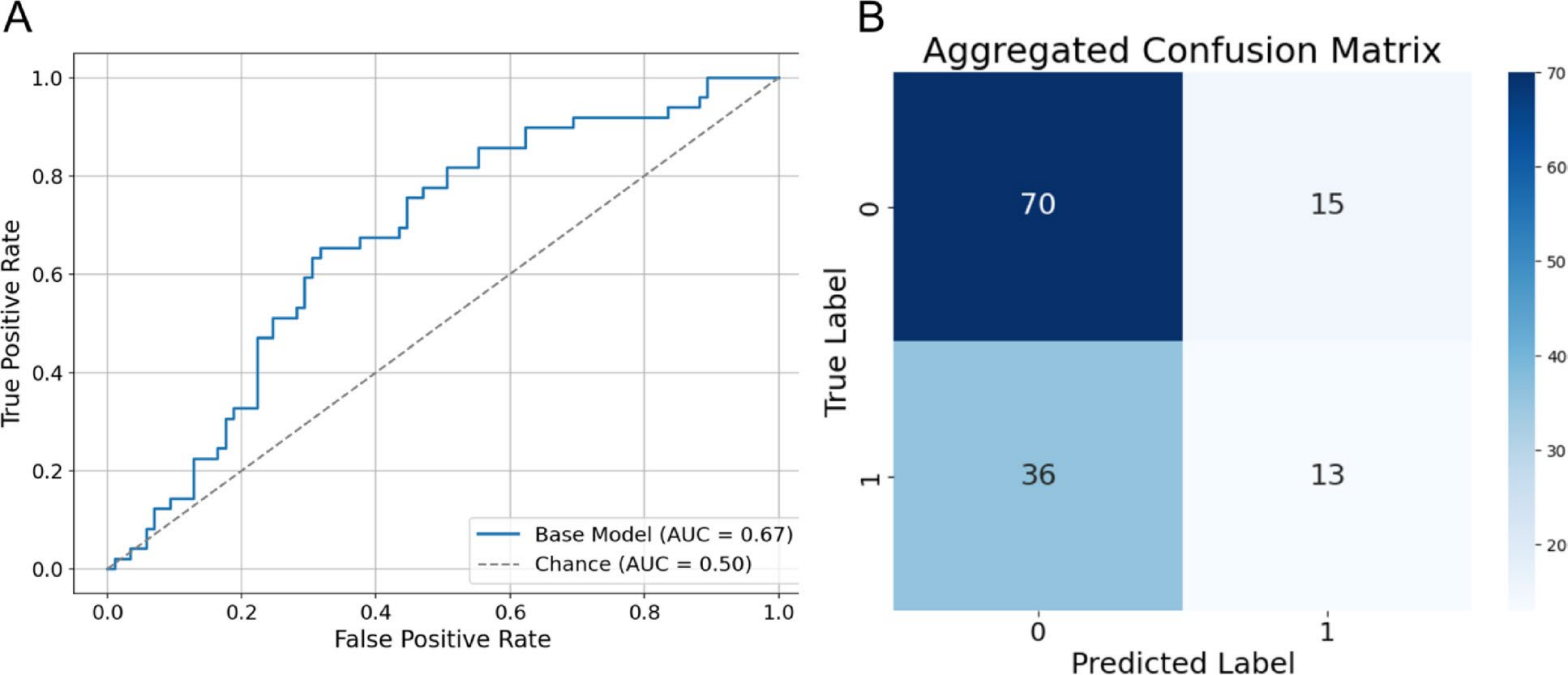
Classification performance of RP-ViT on base (unexpanded) color fundus photos. **A** Receiver Operating Characteristic (ROC) and **B** Confusion matrix for RP-ViT in distinguishing autosomal vs. x-linked RP inheritance patterns.

We first evaluated the diagnostic performance of ViT on color fundus photos affected by RP (RP-ViT). For the purpose of binary model classification, we combined the four inheritance modes into two classes: autosomal (AR and AD) and X-linked (XR and XLC). When trained on the base (unexpanded) dataset, RP-ViT achieved a pooled AUC of 0.67, mean accuracy of 0.62 ± 0.05, and mean specificity of 0.55 ± 0.07 (Fig. 5; Table 2).

### Synthetic data generation

Fréchet Inception Distance (FID) analysis was used to evaluate the realism of synthetic images generated by different expansion strategies. VAE-based methods produced substantially lower FID scores compared to diffusion and GAN approaches for both inheritance groups (Table 3). For the AD/AR cohort, VAE Gen 2 achieved the lowest FID score (42.34), followed by VAE Gen 1 (42.80), whereas diffusion (125.08) and GAN (194.52) produced markedly higher FIDs. Similarly, in the XL/XLC cohort, VAE Gen 1 yielded the lowest FID score (49.96), outperforming VAE Gen 2 (57.81), diffusion (136.44), and GAN (219.88). Overall, VAE-based expansions generated synthetic images most closely aligned with the distribution of real fundus images.

**Table 3:** Fréchet Inception Distance (FID) comparison across synthetic expansion strategies. FID values are reported using an ImageNet pretrained feature extractor.

Inheritance Type	Expansion Strategy	FID (ImageNet)
AD/AR	VAE Gen 1	42.80
AD/AR	VAE Gen 2	42.34
AD/AR	Diffusion	125.08
AD/AR	GAN	194.52
XL/XLC	VAE Gen 1	49.96
XL/XLC	VAE Gen 2	57.81
XL/XLC	Diffusion	136.44
XL/XLC	GAN	219.88

**Fig. 6:**
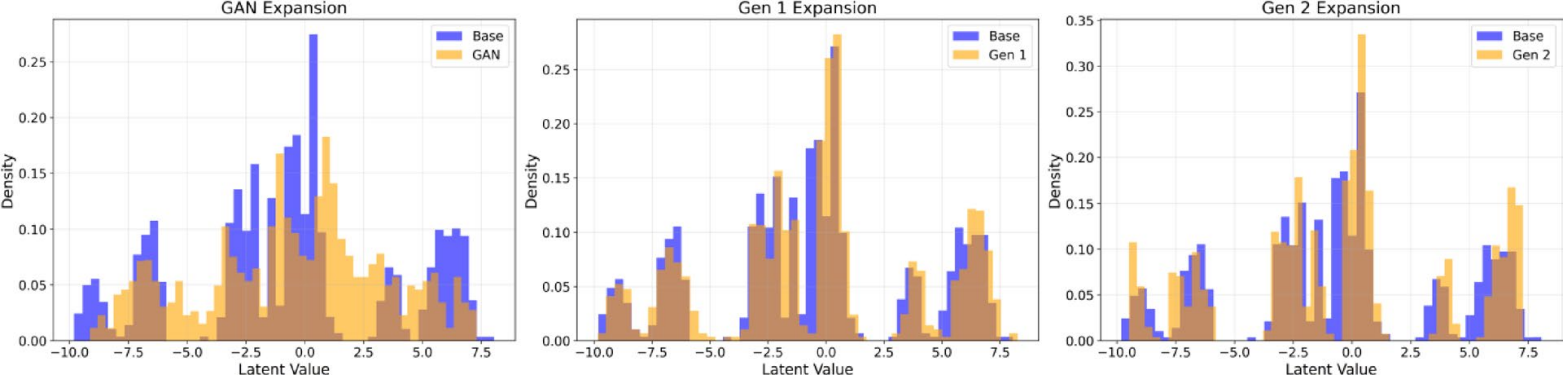
Latent Embedding Distribution. Comparison of density distributions for original data (blue) and expanded data (orange) across GAN (left), VAE Gen 1 (middle) and VAE Gen 2 (right) mixing strategies. The substantial overlap across the latent spectrum indicates that the VAE synthetic samples remain within the original latent distribution, avoiding global distribution shifts or significant out-of-distribution artifacts.

The distribution of latent embeddings was also analyzed to assess how different synthetic expansion strategies influenced the learned feature space (Fig. 6). Compared with the original dataset, GAN-generated samples showed noticeable shifts in the latent distribution, indicating divergence from the underlying feature representation of real images. In contrast, VAE-based expansions demonstrated greater alignment with the latent distribution of the original dataset. Both VAE Gen 1 and Gen 2 maintained similar embedding patterns to the original data, with Gen 2 showing broader coverage of the latent space while preserving the overall distribution structure. These findings suggest that VAE-based expansion produces synthetic samples that remain closer to the original data manifold, while simultaneously increasing representation diversity within the learned feature space.

### Autoencoder enhancement of ViT-based classification of RP inheritance mode

#### Random noise expansion (Gen 1)

**Fig. 7:**
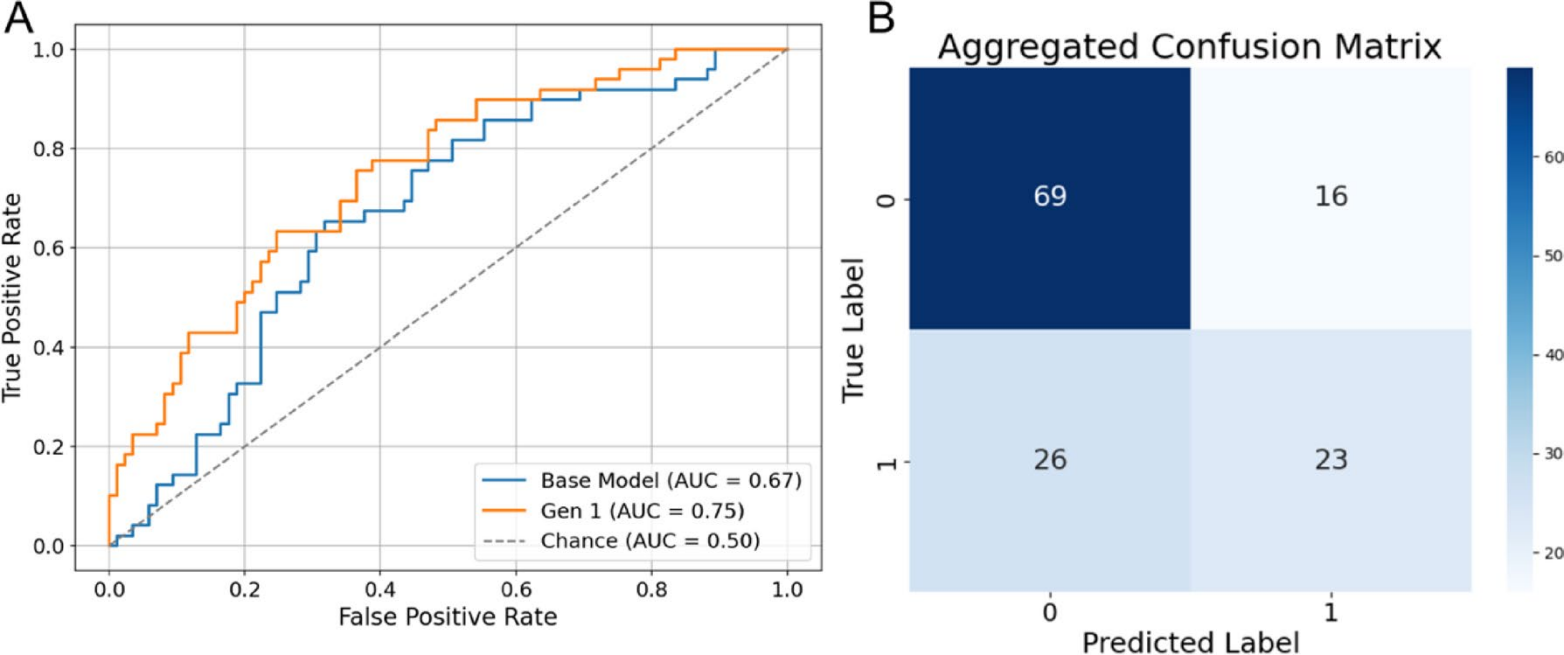
Classification performance of RP-ViT on Gen1-expanded color fundus photos. **A** Receiver Operating Characteristic (ROC) and **B** Confusion matrix for RP-ViT in distinguishing autosomal vs. x-linked RP inheritance patterns.

We trained and evaluated RP-ViT using a two-fold augmented dataset with synthetic images produced by the first-generation VAE method. Gen 1 synthetic data enhancements improved pooled AUC to 0.75, mean accuracy to 0.69 ± 0.05, and mean specificity to 0.64 ± 0.07 (Fig. 7; Table 4).

#### Pair-wise combinatorial expansion (Gen 2)

**Fig. 8:**
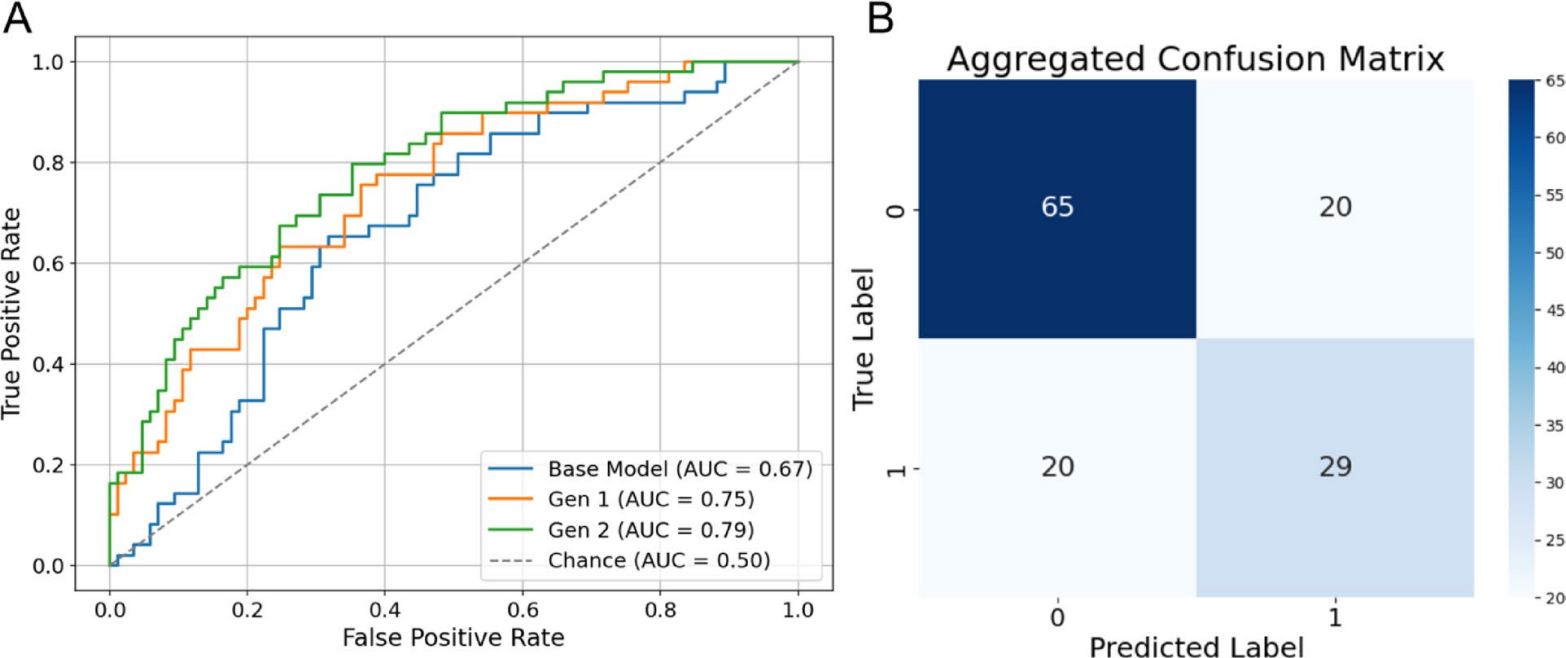
Classification performance of RP-ViT on Gen2- expanded color fundus photos. **A** Receiver Operating Characteristic (ROC) and **B** Confusion matrix for RP-ViT in distinguishing autosomal vs. x-linked RP inheritance patterns.

We evaluated RP-ViT trained on a combinatorial pair-wise expanded dataset enhanced with synthetic images produced by the Gen 2 VAE method. This framework outperformed both the base model and Gen1-expanded RP-ViT models on all measured metrics, with a pooled AUC of 0.79, mean accuracy of 0.71 ± 0.10, and mean specificity of 0.68 ± 0.10 (Fig. 8; Table 4).

**Table 4:** Classification performance metrics of RP-ViT on base (unexpanded) and synthetically enhanced (Gen 1, Gen 2) datasets. Standard deviation ranges are bracketed (−1 SD, + 1 SD).

Expansion	AUC	Pooled AUC	Accuracy	Specificity
Base	0.70 (0.64, 0.76)	0.67	0.62 (0.57, 0.67)	0.55 (0.48, 0.62)
Random (Gen 1)	0.76 (0.68, 0.84)	0.75	0.69 (0.64, 0.74)	0.64 (0.57, 0.71)
Pair-wise (Gen 2)	0.79 (0.68, 0.90)	0.79	0.71 (0.61, 0.81)	0.68 (0.58, 0.78)

Pooled AUC refers to AUC calculated from combined classifications across all folds.

To further characterize the effect of age on classification performance, we conducted age-matched subgroup analyses restricted to bins in which autosomal and X-linked cases overlapped (Table 5). In the 10–20 year bin, class-specific precision was 1.00 for autosomal-dominant and X-linked. In the 50–60 year bin, precision remained high for autosomal-dominant (0.82) and X-linked (1.00). Collectively, these results suggest that RP-ViT leverages inheritance pattern-specific retinal features that remain detectable when constrained to age-matched subsets.

**Table 5:** Age-matched subgroup analyses of classification performance of RP-ViT on Gen2-expanded color fundus photos.

Age Range (years)	Class	Precision	F1 Score
10–20	AD/AR	1.00	1.00
10–20	XL/XLC	1.00	1.00
50–60	AD/AR	0.82	0.78
50–60	XL\XLC	1.00	1.00

**Fig. 9:**
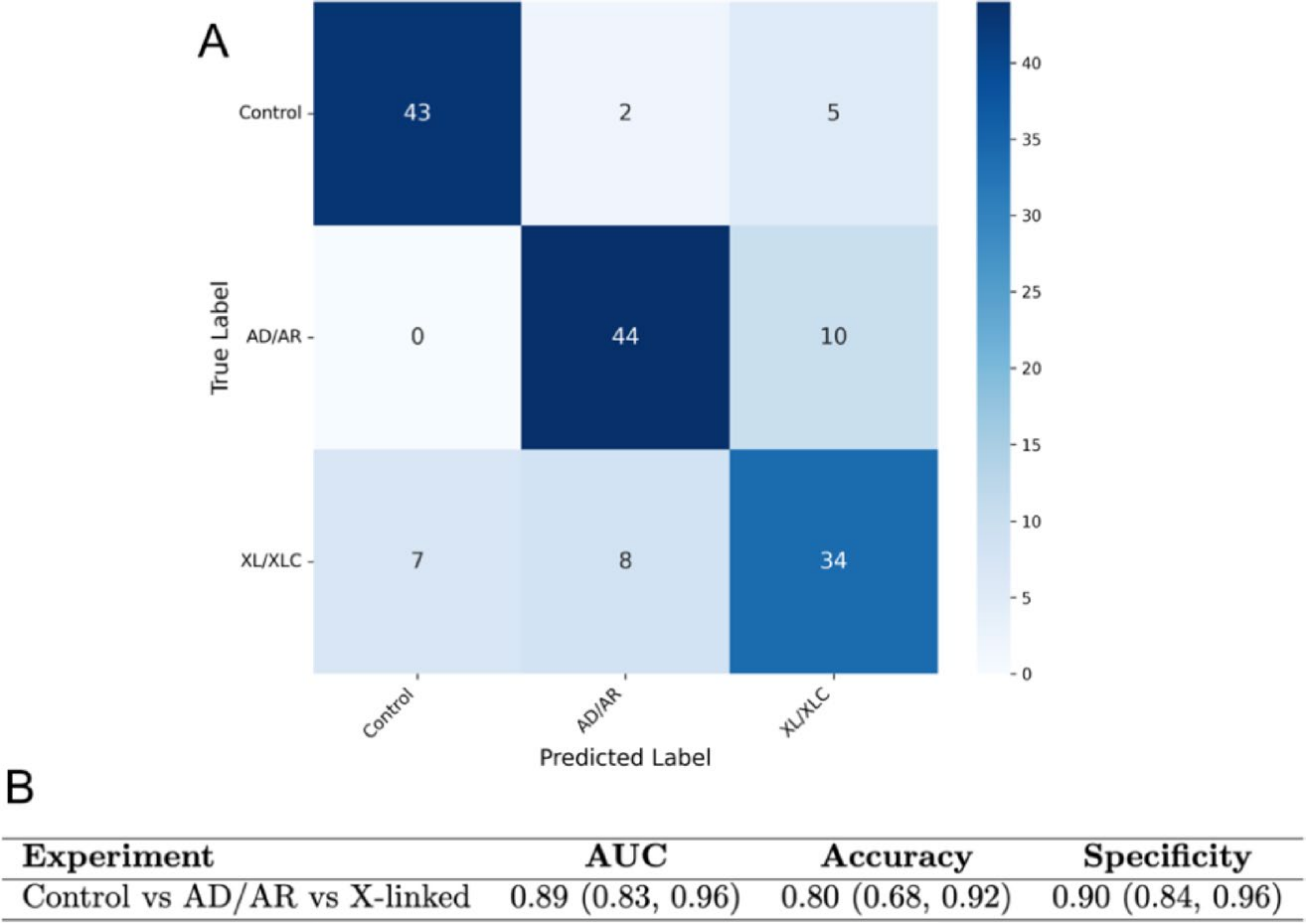
Classification performance of 3-class RP-ViT on Gen 2- expanded color fundus photos. **A** Confusion matrix **B** Performance metrics. For RP-ViT in distinguishing age-matched control vs. AD/AR RP vs. x-linked RP fundus photos. Standard deviation ranges are bracketed (−1 SD, + 1 SD). Finally, we evaluated RP-ViT’s ability to distinguish age-matched normal controls from autosomal RP vs. X-linked RP. When trained on Gen2-expanded color fundus photos, this 3-class variation of RP-ViT achieved an AUC of 0.89, accuracy of 0.80, and specificity of 0.90.

## Discussion

Deep learning systems have achieved near-expert performance in detecting prevalent retinal disorders—most notably diabetic retinopathy and age-related macular degeneration—powered by the vast imaging datasets generated through population-wide screening initiatives^47–49^. Yet their translation into subspecialty clinics has been limited, with few algorithms undergoing rigorous, real-world validation in expert settings where diagnostic subtleties matter most^50,51^. The gap widens further for rare inherited disorders such as retinitis pigmentosa, where patient numbers are small and existing datasets fall several orders of magnitude below typical deep-learning requirements. It thus remains an open question for the field whether AI models trained on such limited cohorts can deliver actionable insights.

In this study, we report on the application of generative AI and deep learning to classify genetic inheritance patterns in rare retinal diseases. Specifically, we demonstrate the feasibility of using a vision transformer (ViT) to identify modes of inheritance from retinitis pigmentosa (RP) fundus images. The selection of a ViT architecture is supported by a comparative analysis against ResNet (Table 2). While both architectures achieved comparable performance on the original dataset (both initialized with ImageNet-pretrained weights and trained with identical optimization settings), ViT exhibited a more substantial performance gain following data expansion. This is consistent with the observation that ViTs scale more effectively as the training set size increases, thereby better leveraging our synthetic samples.

Several synthetic data generation paradigms were evaluated: GAN, diffusion and VAE. Based on FID analysis (Table 3) and distribution of latent embeddings (Fig. 6), VAE was selected as the data expansion platform. We introduced both random-noise (Gen 1) and pair-wise combinatorial (Gen 2) expansions of training data. While both Gen 1 and Gen 2 VAE expansions improve ViT performance, Gen 2 offers a greater range of quantity and diversity in its synthetic data, leading to improvements beyond Gen 1 methods. By generating synthetic data via the fusion of real patient images rather than using GAN methods, we minimize hallucination and maximize clinical fidelity and relevance of the resulting synthetic training data. To evaluate how these latent mixing strategies affect the learned representations, we analyzed the distribution of latent embeddings for both the original and expanded datasets (GAN, VAE Gen 1, and VAE Gen 2) (Fig. 6). In contrast to GAN, both VAE Gen 1 and VAE Gen 2 expanded-data embeddings show substantial overlap with the original-data embeddings, suggesting that the proposed mixing strategies generate samples that remain largely within the latent distribution learned from real images, rather than producing a pronounced global shift in the latent representation. Together, these findings indicate that VAE-based expansion strategy increases dataset diversity while preserving the underlying distribution of real fundus images, supporting its suitability for data augmentation in our setting.

Our findings introduce two important concepts in clinical AI methodology: first, the ability to identify hidden patterns that correlate with inheritance mode and are invisible to human detection, and second, an encoder-decoder generative AI approach to alleviate scarce-data limitations of rare diseases. To our knowledge, this is the first report to use deep learning to classify images by mode of inheritance in retinitis pigmentosa (RP). While the model is capable of distinguishing age-matched normal controls vs. RP eyes in a multi-class classifier (Fig. 9), we recommend a binary classification approach for inheritance pattern detection in RP fundus photos based on both clinical and model design considerations. Classification of inheritance patterns may provide complementary phenotypic context that can support research workflows and exploratory stratification, particularly in cases where a definitive molecular diagnosis has not been established^52–54^. Distinguishing between X-linked and autosomal inheritance in IRDs may help contextualize phenotypic presentation, support exploratory predictions about disease progression, and aid in prioritizing diagnostic evaluation, particularly when a clear molecular diagnosis is not available. Of interest, our binary classifier model demonstrated better accuracy compared to a 4-class classifier. We suspect this difference may be due to the inherent limitations of classifiers in handling multiclass problems, as well as the small size of rare disease datasets^55^. Multiclass classifiers are intrinsically more data-hungry, a limitation that becomes pronounced in rare-disease settings where sample counts are low. While our data augmentation pipelines aim to address this data scarcity problem, their benefits, particularly that of Gen 2, scale with the size of the native dataset because each new image arises from two distinct originals. Consequently, the smallest cohorts (e.g. <20 images) still pose a challenge. Ongoing efforts therefore aim to further advance data expansion techniques to enable AI tools that perform robustly even with very small datasets, thereby improving classification accuracy in multi-class problems.

Beyond building a more robust data-enhancement architecture, we anticipate that a discovery-focused approach to AI development will facilitate the identification of previously unrecognized sub-visual biological signatures. Pathology unfolds across many measurement axes (e.g. structural, functional, and molecular), each contributing to a slice of a high-dimensional landscape. Aligned with the emerging field of oculomics, we seek to extract biological and systemic insights from ocular imaging data. By integrating complementary modalities, such as optical-coherence tomography, fundus imaging, and electrophysiology, AI provides a more complete representation of inherited retinal disorders and, accordingly, may improve DL classification performance^56^.

We acknowledge several limitations to our approach. First, we relied on a limited dataset from a single institution. While UCSF is a tertiary referral center, the cohort available for this initial study is nevertheless limited. In particular, our dataset does not include the full differential diagnosis of retinal degeneration. Accordingly, model performance in settings beyond retinitis pigmentosa is unknown, and the risk of misclassification for other etiologies is not assessed in this report. Furthermore, the cohort consists predominantly of patients with advanced disease, therefore, the model is not intended for clinical deployment as a diagnostic tool. Secondly, we acknowledge that the reported performance is insufficient for clinical deployment and should not be interpreted as evidence of clinical readiness. Our objective was instead to evaluate whether deep learning models can learn discriminative signal under extreme data scarcity and whether data-expansion strategies improve model learning under fixed constraints. In this sense, the work serves as a methodological proof-of-concept and a first step toward more robust modeling in severely limited rare-disease datasets. Future works towards achieving clinically meaningful performance, such as predicting prognosis and progression, will incorporate substantially larger and more diverse cohorts, external (ideally prospective) validation, and evaluation at clinically actionable operating points. Furthermore, the autosomal and X-linked groups in our cohort differed significantly in mean age—an expected reflection of the earlier onset typical of X-linked IRDs. While this age disparity presents a potential confounding factor, it simultaneously reflects a genuine clinical distinction in onset and progression between inheritance patterns; the differences we observed likely reflect real differences in disease severity that correlate with inheritance pattern. To further characterize the effect of age on classification performance, we conducted age-matched subgroup analyses restricted to bins in which autosomal and X-linked cases overlapped (Table 5). The high-performance metrics observed in both the younger (10–20 year old) and older (50–60 year old) bins suggest that the proposed framework is detecting meaningful disease-specific signals beyond age-related retinal features. While these results support the presence of phenotype-specific features associated with RP inheritance patterns, larger age-matched cohorts will be necessary to more rigorously assess the relative contributions of age and genotype to model predictions.

Finally, similar to other deep learning models, our method involves a level of complexity that can obscure the interpretability of its decision-making process — making it difficult to determine which features were most relevant to the model predictions. While established interpretability tools exist for earlier architectures such as CNNs, the self-attention mechanisms used in Vision Transformers (ViTs) make it more challenging to generate intuitive or clinically recognizable explanations^57^. To address this limitation, there is ongoing work to develop a multimodal large language–vision model to articulate its visual reasoning for medical image analyses.

In conclusion, this study highlights the potential of deep learning to contribute to the diagnostic workflow for inherited retinal diseases. By integrating VAE-powered data enhancement with ViT-driven binary classification, we report a novel and effective proof-of-concept framework for mitigating the limitations imposed on AI tool development by inherently scarce data and may provide supplemental information about disease severity in resource-limited settings where genetic testing is not available or results are indeterminate. The reported framework enabled classification of RP inheritance mode using a small dataset, fulfilling an important clinical objective and underscoring the potential of AI to enhance healthcare for all patient populations, irrespective of disease prevalence.

## Data Availability

The datasets used and/or analyzed during the current study available from the corresponding author on reasonable request.

## Code availability

The custom code used to generate results central to this study’s claims is publicly available on GitHub at https://github.com/mlrivera123/Deep-Learning-Detection-of-Retinitis-Pigmentosa and has been archived with a DOI at 10.5281/zenodo.19225940.
